# Supplementary material for: Evaluating the effects of community treatment orders (CTOs) in England using the Mental Health Services Dataset (MHSDS): protocol for a national, population-based study
Source: BMJ Open. 2018 Oct 18;8(10):e024193. doi: 10.1136/bmjopen-2018-024193 (PMC6196959; doi:10.1136/bmjopen-2018-024193)
Supplement: Author's manuscript [file bmjopen-2018-024193.draft_revisions.pdf]

BMJ Open is committed to open peer review. As part of this commitment we make the peer review history of every article we publish publicly available.

When an article is published we post the peer reviewers' comments and the authors' responses online. We also post the versions of the paper that were used during peer review. These are the versions that the peer review comments apply to.

The versions of the paper that follow are the versions that were submitted during the peer review process. They are not the versions of record or the final published versions. They should not be cited or distributed as the published version of this manuscript.

BMJ Open is an open access journal and the full, final, typeset and author-corrected version of record of the manuscript is available on our site with no access controls, subscription charges or pay-per-view fees (http://bmjopen.bmj.com).

If you have any questions on BMJ Open's open peer review process please email info.bmjopen@bmj.com

BMJ Open

Evaluating the effects of Community Treatment Orders (CTOs) in England using the Mental Health Services Dataset (MHSDS): Protocol for a national, population-based study

Journal:	*BMJ Open*
Manuscript ID	bmjopen-2018-024193
Article Type:	Protocol
Date Submitted by the Author:	16-May-2018
Complete List of Authors:	Weich, Scott; University of Sheffield, School of Health and Related Research<br>Duncan, Craig; University of Portsmouth, Geography;<br>Bhui, Kamaldeep; Barts and The London School of Medicine & Dentistry Queen Mary University of London, Centre for Psychiatry at the Wolfson Institute of Preventive Medicine<br>Canaway, Alastair; University of Warwick Warwick Medical School<br>Crepaz_Keay, David; Mental Health Foundation<br>Keown, Patrick; Newcastle University Faculty of Medical Sciences, Institute of Neuroscience; Northumberland Tyne and Wear NHS Foundation Trust,<br>Madan, Jason; University of Warwick, Warwick Medical School<br>McBride, Orla; Ulster University, Psychology<br>Moon, Graham; University of Southampton, Geography and Environment<br>Parsons, Helen; University of Warwick, Division of Health Sciences<br>Singh, Swaran; Warwick Medical School, Health Sciences Research Institute<br>Twigg, Liz; University of Portsmouth, Department of Geography
Keywords:	Adult psychiatry < PSYCHIATRY, Quality in health care < HEALTH SERVICES ADMINISTRATION & MANAGEMENT, Health policy < HEALTH SERVICES ADMINISTRATION & MANAGEMENT

SCHOLARONE™
Manuscripts

Evaluating the effects of Community Treatment Orders (CTOs) in England using the Mental Health Services Dataset (MHSDS): Protocol for a national, population-based study

Scott Weich [a*], Craig Duncan [b], Kamaldeep Bhui [c], Alastair Canaway [d], David Crepaz-Keay [e], Patrick Keown [f], Jason Madan [d], Orla McBride [g], Graham Moon [h], Helen Parsons [d], Swaran Singh [d], Liz Twigg [b]

[a] ScHARR
University of Sheffield
Sheffield S1 4DA
United Kingdom
t: +44 (0)114 222 0753/0856
f: +44 (0)114 222 0749
e: s.weich@sheffield.ac.uk
* Corresponding author

[b] Department of Geography
University of Portsmouth
Portsmouth PO1 3HE
United Kingdom

[c] Centre for Psychiatry
Barts and The London School of Medicine & Dentistry
Queen Mary University of London
London EC1M 6BQ
United Kingdom

[d] Warwick Medical School,
University of Warwick
Coventry CV4 7AL
United Kingdom

[e] Mental Health Foundation
1 London Bridge Walk
London SE1 2SX
United Kingdom

[f] Institute of Neuroscience
Newcastle University
Newcastle NE1 7RU
United Kingdom

[g] School of Psychology
Ulster University
Londonderry BT48 7JL
United Kingdom

For peer review only - http://bmjopen.bmj.com/site/about/guidelines.xhtml

[h] Geography and Environment
University of Southampton
Southampton SO17 1BJ
United Kingdom

Keywords: Community treatment orders; Supervised community treatment; Community mental health

Word count: 2,696 words (excluding title page, references, figures and tables)

For peer review only

For peer review only - http://bmjopen.bmj.com/site/about/guidelines.xhtml

Abstract (300 words)

Introduction

Supervised Community Treatment (SCT) for people with serious mental disorders has become accepted practice in many countries around the world. In England, SCT was adopted in 2008 in the form of Community Treatment Orders (CTOs). CTOs have been used more than expected, with significant variations between people and places. There is conflicting evidence about the effectiveness of SCT; studies based on randomised controlled trials (RCTs) have suggested few positive impacts, while those employing observational designs have been more favourable. Robust population-based studies are needed, because of the ethical challenges of undertaking further RCTs and because variation across previous studies may reflect the effects of socio-spatial context on SCT outcomes. We aim to examine spatial and temporal variation in the use, effectiveness and cost of CTOs in England through the analysis of routine administrative data.

Methods and analysis

Four years of data from the Mental Health Services Dataset (MHSDS) will be analysed using multilevel models (MMs). Models based on all patients eligible for CTOs will be used to explore variation in their use. A subset of CTO-eligible patients comprising a treatment group (CTO-patients) and a matched control group (non-CTO patients) will be used to examine variation in the association between CTO use and study outcomes. Primary outcome will be total time in hospital. Secondary outcomes will include time to first re-admission and mortality. Outputs from these models will be used to populate predictive models of health care resource use.

For peer review only - http://bmjopen.bmj.com/site/about/guidelines.xhtml

Ethics and dissemination

Ethical approval has been granted by the NHS Data Access and Advisory Group and Warwick Medical School. To ensure patient confidentiality and to meet data governance requirements, analyses will be carried out in a secure micro-data laboratory using de-identified data. Study findings will be disseminated through academic channels and shared with mental health policy makers and other stakeholders.

For peer review only

For peer review only - http://bmjopen.bmj.com/site/about/guidelines.xhtml

Strengths and limitations of this study

- This will be the largest and most complete study of its kind and the national representativeness of the study sample, deriving from routine clinical activity, is a major strength.
- The study design reflects the range of real world settings in which mental health services are delivered and patients live in England.
- The use of multilevel models will allow us to estimate variation in CTO use and outcome at individual-, area- and service-level and to establish who CTOs work for and in what circumstances.
- Study limitations include confounding by indication and bias arising from missing data within the routine administrative data source.

For peer review only

For peer review only - http://bmjopen.bmj.com/site/about/guidelines.xhtml

Introduction

Community Treatment Orders (CTOs), a form of Supervised Community Treatment (SCT), were introduced to the Mental Health Act (2007) (MHA) in England and Wales in 2008 and allow certain patients detained in hospital to continue treatment in the community following discharge under specific conditions. To be eligible for a CTO, a patient must be detained in hospital under the MHA on a Section that allows for compulsory treatment. The most common inpatient treatment orders are Sections 3 and 37, the former being a civil order and the latter a forensic, court-imposed equivalent for mentally ill offenders. A CTO provides the clinical team the power to recall the patient to hospital after discharge.

At CTO initiation (the point of discharge from inpatient care) there are two mandatory conditions, namely that the person subject to a CTO makes themselves available for assessment for its renewal and (where necessary) to be seen by an approved second opinion doctor (SOAD) for the purposes of assessing proposed treatment plan should the patient lack capacity in respect of their treatment. Discretionary conditions can also be specified which most often include treatment (i.e. medication) adherence and engagement with services (e.g. attending for appointments with professionals). CTOs do not permit compulsory (i.e. forcible) treatment in the community,[1] but those who default from these conditions may be recalled to hospital for assessment if there are grounds to suspect deterioration in their mental health and/or risk to the patient or others. Where this assessment indicates the need for further treatment, the CTO is revoked and the original treatment order applies.

There were two motivations for introducing CTOs in England and Wales[2]: to reduce 'revolving door' admissions associated with non-adherence with care by a small group of patients, and

For peer review only - http://bmjopen.bmj.com/site/about/guidelines.xhtml

to allow treatment in the least restrictive setting, in accordance with the Human Rights Act (1988). While it was originally envisaged that around 10% of eligible patients would be placed on CTOs, the figure is currently closer to 30%.[3] Since more CTOs are issued than are ended each year, the number of patients subject to CTOs has increased over time. Almost five thousand CTOs were issued in 2016-17, with use varying between demographic groups: rates of CTO use for males (11.4 per 100,000 population) were almost twice the rate for females (6.6 per 100,000 population), and the rate for Black and Black British people (60.1 per 100,000 population) was almost nine times the rate for those of White ethnicity (6.8 per 100,000 population).[4] Use also varies between National Health Service (provider) Trusts.[5,6]

SCT has been adopted in over 75 other jurisdictions around the world,[7] although it is used differently in different places. This is highlighted by its different names, including 'involuntary outpatient commitment', 'assisted outpatient treatment', 'supervised discharge', 'compulsory community treatment' and 'compulsory ambulatory treatment'.[8] In some places, SCT is initiated only by courts, while elsewhere, as in England, it remains a matter exclusively for health and social care providers, albeit within a framework prescribed by Statute and subject to legal review. There are also differences between countries in the organisation and funding of mental health services, including separation of in-patient from community mental health teams and variations in the nature and availability of care for people with severe mental illness. Generalising findings between countries is therefore problematic.

Despite widespread use, SCT remains controversial. In part, this is because patients and carers see it as coercive, raising fears of negative effects on therapeutic relationships.[9] This disquiet has been heightened by conflicting research evidence about effectiveness.[10] There

For peer review only - http://bmjopen.bmj.com/site/about/guidelines.xhtml

have been only three RCTs of CTOs, two in the US and one in the UK. None demonstrated significant benefit of CTOs in terms of hospital readmission, social functioning, quality of life, mental state or offending.[11-13] Randomised trials of CTOs face particular ethical and practical challenges (including relatively brief duration of follow-up), and these limit their utility.[14] In critically appraising the two US trials, Kisely and colleagues highlighted small sample sizes, the court-imposed nature of compulsory community treatment and the exclusion of patients with a history of violence. The OCTET trial in England experienced several methodological limitations, including the involuntary nature of the control condition, high rates of non-participation and patients who moved between trial arms after randomisation.[15-17]

Apart from the inevitable unblinding of participants and clinicians, it is impossible to disentangle the effects of legal compulsion from the vigilance and intensive care associated with CTO implementation.[8] Further trials of CTOs are unlikely, given ethical challenges inherent in randomising those who may lack insight, and who may be dangerous to themselves or others, to legal restriction versus truly voluntary care.[18]

The results of observational studies are less consistent, with some showing decreased admission rates and reduced bed use among those subject to SCT.[2,8,14,19] After excluding uncontrolled studies, observational research is characterised by matched cohort designs and before and after studies, with outcomes generally (but not invariably) assessed over two years or less. More recently, findings have emerged which suggest that patients on CTOs may experience better physical health, and reduced mortality.[20,21]

For peer review only

Population-based observational studies using routine clinical and administrative data allows analysis of variation in SCT use and outcome to be studied.[10,22] We will use four years of data from the Mental Health Services Dataset (MHSDS) to address the following aims: (1) to examine spatial and temporal variation in the use of CTOs; (2) to estimate associations between CTO use and patient outcomes; (3) to explore variation in these associations between patients, places and providers, establishing when, where and for whom CTOs may be effective; and (4) to estimate the financial costs and benefits associated with CTOs.

Methods and analysis

Data sources and study outcomes

The use of CTOs in England is recorded as part of a mandatory administrative dataset, the Mental Health Services Dataset (MHSDS), formerly known as the Mental Health Minimum Dataset (MHMDS), and then the Mental Health and Learning Disabilities Dataset (MHLDDS).[23] MHSDS collates monthly returns from health service providers on all patients in contact with secondary mental health services provided and/or funded by NHS England. This includes voluntary and involuntary in-patient treatment, out-patient attendance, day treatment and other episodes of secondary mental health care.

MHSDS data are based on spells of care for individual patients. Since 2011, the dates on which specific care spells start and end have been included. Care spell dates can therefore be used to determine duration of CTO use, and hospital (re-)admissions. Four years of MHSDS data will be used covering the period 2011/12 to 2014/15, hereafter referred to as the study period. MHSDS records contain a unique patient identifier code which will be used to link patient care spells across the study period. This identifier will also be used to link patient

For peer review only - http://bmjopen.bmj.com/site/about/guidelines.xhtml

records to the Office for National Statistics' (ONS) Mortality Database, to ascertain patient deaths. Access to, and linkage of, these different administrative data sets will be facilitated by the ESRC Administrative Data Research Network.

Covariates

MHSDS provides data on a range of patient characteristics, including age, sex and ethnicity. Data on clinical characteristics are also available such as diagnosis, care clusters (groupings of patients with similar needs and problem severities)[24] and Health of the Nation Outcome Scales (outcome ratings for a range of health and social domains).[25] Additional patient characteristics such as marital, employment and accommodation status may be present in the dataset, although previous research noted high levels of missing data.[26]

MHSDS patient records also include several spatial/service setting identifiers (Table 1). These will be used to link patient records to data on factors shown by previous studies to influence mental health outcomes at local area and service provider level. Specifically, English Indices of Deprivation 2015 will provide information about local area socio-economic characteristics while ONS 2011 Census data will be used to characterise local populations, including through the use of population density and ethnic composition data.[27,28]

Data analysis

As the overarching aim of the study is to examine spatial and temporal variation in CTO use and outcome, multilevel models (MMs) will be used.[29] MMs allow variation in outcomes to be apportioned to the appropriate spatial level (e.g. between patients, local areas and service settings) as well as estimating associations with individual-, area- and service-level

For peer review only - http://bmjopen.bmj.com/site/about/guidelines.xhtml

characteristics.[30] Since the local areas that patients live in do not nest hierarchically within the health service settings where they receive care (and vice versa), cross-classified multilevel models (CCMMs) will be used (Figure 1).[31]

Analysis will be conducted in three stages. First, CCMMs based on all patients eligible for CTOs will be used to explore spatial and temporal variation in the use of CTOs. CTO use will be considered as a binary outcome (patients subject to a CTO during the study period versus those who were not) and analyses will be undertaken using logistic multilevel regression. Models will estimate the variation at each level in the risk of being on a CTO and assess the extent to which any variation in this outcome is associated with patient, local area and health service characteristics. We will also investigate the duration of time spent subject to CTO (in days). Poisson/negative binomial link function CCMMs will be used and will include offset variables to take account of different observation times for patients.[32]

Next, to examine associations between CTO use and a range of outcomes, a cohort of patients will be constructed consisting of a treatment group (those who have been on a CTO) and a matched control group (those who are eligible but who have not been on a CTO). To prevent bias arising from secular changes in clinical practice, we will frequency-match these groups according to the start date of the CTO (CTO group) and the date of discharge from hospital following detention under a CTO-eligible section of the Mental Health Act (non-CTO group). Models based on the cohort of matched patients will be used to test associations between SCT use and patient outcomes: total time spent in hospital, time to first re-admission and mortality. For total time spent in hospital, count models based on Poisson or negative binomial distributions will be estimated. For time to first re-admission, discrete-time survival

For peer review only - http://bmjopen.bmj.com/site/about/guidelines.xhtml

analysis models will be estimated. For mortality, binary response models based on the logistic distribution will be estimated. To control for confounding by indication, we will adjust analyses for the propensity of being placed on SCT.[33] CCMMs based on this cohort will be used to test for the overall 'average' effect of CTO on each of the study outcomes. The models will also assess whether there is significant spatial and temporal variation around these average effects and estimate the extent to which this is associated with patient, local area and health service characteristics.

Finally, we will use outputs from our CCMMs to populate predictive models of health care resource use incorporating the predicted impact of CTOs. We will model differences in treatments costs (e.g. bed days and number of contacts with mental health staff) between patients who are and are not on CTOs. We will also quantify the administrative burden of CTOs, using protocols for mandated governance processes (e.g. Mental Health Act Review Tribunals (MHRTs) and Second Opinion Approved Doctors (SOADs)) that apply to the use of CTOs. Projected costs associated with different plausible future trajectories of CTO use will be calculated and a probabilistic sensitivity analysis will be presented for each projection based on estimates of parameter uncertainty derived from the CCMMs.

CCMMs will be estimated in MLwIN using Markov Chain Monte Carlo (MCMC) Bayesian methods.[34] This approach is suited to the complexity of the MHSDS and will provide unbiased measures of variation in CTO use and outcome, and associations with patient, local area and health setting characteristics. MCMC modelling diagnostics, together with measures of multilevel variation, will be used to evaluate different models to quantify where, and for whom, associations and variation occurs.[35-37]

For peer review only - http://bmjopen.bmj.com/site/about/guidelines.xhtml

For peer review only

Patient and Public Involvement

Two reference groups will provide guidance to the project to maximise its relevance and impact. The first group will consist of mental health service users and carers and will include people with lived experience of being subject to CTOs or of caring for someone who has. Participants will be recruited by the Mental Health Foundation (MHF), the largest mental health policy, research and service improvement charity in England. Recruitment will ensure diversity of age, gender and ethnicity. The second group will consist of mental health professionals and will include managers and clinicians. The reference groups will help us ensure that analyses and dissemination are relevant to the needs of stakeholders.

Dissemination

CTOs are used more often than originally anticipated in England. Although many clinicians continue to see their value[38], uncertainty and concern about their use has been expressed by regulators and legislators.[3,39] Both have called for the sort of nuanced evidence about the benefits and hazards of CTO use in real world settings which can only come from large-scale, population-based, observational research. The purposes of this study are to describe practice in respect of CTO use and duration, to estimate associations with, and spatial variation in, outcomes, and to understand where, and for whom, CTOs might be of benefit. We will also model the economic costs and benefits of current patterns of CTO use.

This will be the largest and most complete study of its kind in England. The national scope and representativeness of the study sample, deriving from routine clinical activity, is a major strength, as is duration of follow-up. Study limitations are those that affect observational and secondary research, particularly confounding by indication, residual confounding and bias.

For peer review only - http://bmjopen.bmj.com/site/about/guidelines.xhtml

Issues of data quality (including missing data) are important sources of the latter. [40] The study findings will be of direct policy relevance, and we plan to share them directly with those bodies working in this area at the earliest opportunity.

For peer review only

For peer review only - http://bmjopen.bmj.com/site/about/guidelines.xhtml

Table 1: *Spatial/service setting identifiers present in the Mental Health Services Dataset (MHSDS)*

**Spatial/Service Setting Identifier**	**Description**
Lower Layer Super Output Area (LSOA)	Local Area of Residence, based on 2001 Census boundaries, typically containing 672 households and 1,614 residents
GP Practice	Primary Care Provider
NHS and Independent Sector Provider Trust	Provider of Secondary Mental Health Care
Primary Care Trust (PCT)	Commissioner of Secondary Mental Health Care

For peer review only

For peer review only - http://bmjopen.bmj.com/site/about/guidelines.xhtml

Authors' contributions

SW, KB, DC-K, PK, JM, OM, GM, HP, SS, LT had the original idea for the study and were responsible for study hypotheses, design and data specification. CD was responsible for drafting the manuscript. SW has overall responsibility for the conduct of the study. All authors contributed, read and approved the final manuscript.

Funding

The work was supported by the National Institute for Health Research Health Services and Delivery Research Programme (project number 14/52/40).

Competing interests

None.

Ethics approval

The study is approved by the NHS Data Access and Advisory Group and Warwick Medical School Biomedical Research Ethics Committee.

For peer review only - http://bmjopen.bmj.com/site/about/guidelines.xhtml

References

1 Care Quality Commission. *Monitoring the Mental Health Act in 2011/12*. 2013.

2 Churchill R, Owen G, Singh S et al. *International experiences of using community treatment orders*. London: Department of Health. 2007.

3 House of Commons Health Committee. *Post-Legislative Scrutiny of the Mental Health Act 2007: First Report of Session 2013–14*. London: House of Commons. 2013.

4 NHS Digital. *Mental Health Act Statistics, Annual Figures 2016/17 Experimental Statistics*. 2017

5 Trevithick L, Carlile J, Nodiyal S, Keown P. Community treatment orders: an analysis of the first five years of use in England. *The British Journal of Psychiatry*. 2018; 212(3):175-9.

6 NHS Digital. *Inpatients formally detained in hospitals under the Mental Health Act 1983, and patients subject to supervised community treatment Uses of the Mental Health Act: Annual Statistics, 2015/16*. 2016.

7 Rugkåsa J. Effectiveness of community treatment orders: the international evidence. *The Canadian Journal of Psychiatry* 2016; 61: 15-24.

8 O'Brien AJ, McKenna BG, Kydd RR. Compulsory community mental health treatment: Literature review. *International J Nurs Stud*. 2009; 46:1245-55.

9 Light E. The epistemic challenges of CTOs. *Psychiatric Bulletin* 2014; 38: 6-8.

10 Kisely S. Assessing the effectiveness of compulsory community treatment. In Molodynski A, Rugkåsa J, Burns T eds. *Coercion in Community Mental Health Care: International Perspectives.* Oxford 2016: 75-94.

11 Swartz MS, Swanson JW, Wagner HR et al. Can involuntary outpatient commitment reduce hospital recidivism?: findings from a randomized trial with severely mentally ill individuals. *American Journal of Psychiatry* 1999; 156: 1968-1975.

12 Steadman, HJ, Gounis K, Dennis D et al. Assessing the New York City involuntary outpatient commitment pilot program. *Psychiatric Services* 2001; 52: 330-336.

13 Burns T, Rugkåsa J, Molodynski A, et al. Community treatment orders for patients with psychosis (OCTET): a randomised controlled trial. *The Lancet* 2013; 381: 1627-1633.

14 Kisely S, Campbell LA, Scott A et al. Randomized and non-randomized evidence for the effect of compulsory community and involuntary out-patient treatment on health service use: systematic review and meta-analysis. *Psychological Medicine* 2007; 37: 3-14.

15 Curtis D. OCTET does not demonstrate a lack of effectiveness for community treatment orders. *Psychiatric Bulletin* 2014; 38: 36-39.

16 Mustafa FA. On the OCTET and supervised community treatment orders. *Med Sci Law* 2014; 54: 116–117.

17 Owen A, Sood L. OCTET does not prove community treatment orders are ineffective. *The Lancet Psychiatry* 2015; 2: 373-375.

18 Treatment Advocacy Center. *No Relevance to Assisted Outpatient Treatment in the OCTET Study of English Compulsory Treatment*. 2014.

19 Maughan D, Molodynski A, Rugkåsa J et al. A systematic review of the effect of community treatment orders on service use. *Social psychiatry and psychiatric epidemiology* 2014; 49: 651-663.

For peer review only - http://bmjopen.bmj.com/site/about/guidelines.xhtml

20 Nakhost A, Perry JC, Frank D. Assessing the outcome of CTOs on management of psychiatric patients. *Can J Psychiatry*. 2012; 57:359-65.

21 Kisely S, Xiao J, Lawrence D, Jian L. Is the effect of compulsory community treatment on preventable deaths from physical disorders mediated by better access to specialized medical procedures? *Can J Psychiatry*. 2014; 59:54-8.

22 Johnson S. Can we reverse the rising tide of compulsory admissions? *Lancet* 2013; 1603-1604.

23 The NHS Information Centre. *MHMDS Statistics: Data Quality and Methodology*. 2011.

24 NHS England. Mental Health Clustering Booklet v5.0, 2016/17. https://assets.publishing.service.gov.uk/government/uploads/system/uploads/attachment_data/file/499475/Annex_B4_Mental_health_clustering_booklet.pdf

25 Royal College of Psychiatrists. Health of the Nation Outcomes Scales (HoNOS) Glossary, 2018. https://www.rcpsych.ac.uk/pdf/CALC_HoNOS2_FinalVersion_2017A.pdf

26 Weich, S., McBride, O., Twigg, L., et al. Variation in compulsory psychiatric inpatient admission in England: a cross-classified, multilevel analysis. *The Lancet Psychiatry*. 2017; 4:619-626.

27 Ministry of Housing, Communities and Local Government. *English Indices of Deprivation*. 2015.

28 Office for National Statistics. *2011 Census aggregate data*. 2016.

29 Snijders TA, Bosker R. *Multilevel Analysis: An Introduction to Basic and Advanced Multilevel Modeling (2nd Edition)*. London: Sage. 2012.

30 Merlo J. Multilevel analytical approaches in social epidemiology: measures of health variation compared with traditional measures of association. *Journal of epidemiology and community health* 2003; 57: 550-552.

31 Fielding A, Goldstein H. *Cross-Classified and Multiple Membership Structures in Models: An Introduction and Review. Research Report RR791 for DfES.* London: Department for Education and Skills; 2006.

32 Austin, PC, Stryhn H., Leckie G. and Merlo J. Measures of clustering and heterogeneity in multilevel Poisson regression analyses of rates/count data. *Statistics in Medicine 2018; 37:* 572-589.

33 Austin PC. An introduction to propensity score methods for reducing the effects of confounding in observational studies. *Multivariate behavioral research* 2011; 46: 399-424.

34 Browne WJ *MCMC Estimation in MLwiN v2.1.* Centre for Multilevel Modelling, University of Bristol. 2009.

35 Raftery AE, Lewis SM. [Practical Markov Chain Monte Carlo]: Comment: One Long Run with Diagnostics: Implementation Strategies for Markov Chain Monte Carlo. *Stat Sci* 1992; 7(4):493–7.

36 Spiegelhalter DJ, Best NG, Carlin BP, van der Linde A. Bayesian measures of model complexity and fit. *J R Stat Soc Ser B* 2002; 64: 583–639.

37 Merlo J, Ohlsson H, Lynch KF, et al. Individual and collective bodies: using measures of variance and association in contextual epidemiology. *Journal of epidemiology and community health* 2009; 63: 1043-1048.

38 DeRidder R, Manning C, Molodynski A et al. Community treatment orders in the UK 5 years on: a repeat national survey of psychiatrists. *BJPsych Bull* 2016; 40: 119-123.

39 Care Quality Commission. *Monitoring the Mental Health Act in 2012/13*. 2014.

For peer review only - http://bmjopen.bmj.com/site/about/guidelines.xhtml

40 Stuart EA, Azur M, Frangakis C et al. Multiple imputation with large data sets: a case study of the Children's Mental Health Initiative. *American journal of epidemiology* 2009; 169: 1133-9.

For peer review only

For peer review only - http://bmjopen.bmj.com/site/about/guidelines.xhtml

Figure 1: *An illustration of cross-classified multilevel data*

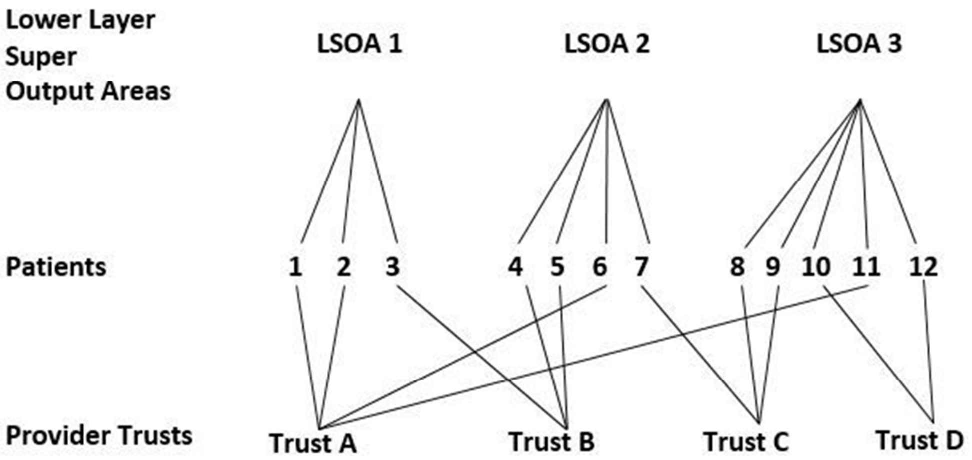

53x31mm (300 x 300 DPI)

review only

BMJ Open

Evaluating the effects of Community Treatment Orders (CTOs) in England using the Mental Health Services Dataset (MHSDS): Protocol for a national, population-based study

Journal:	*BMJ Open*
Manuscript ID	bmjopen-2018-024193.R1
Article Type:	Protocol
Date Submitted by the Author:	13-Jun-2018
Complete List of Authors:	Weich, Scott; University of Sheffield, School of Health and Related Research<br>Duncan, Craig; University of Portsmouth, Geography;<br>Bhui, Kamaldeep; Barts and The London School of Medicine & Dentistry Queen Mary University of London, Centre for Psychiatry at the Wolfson Institute of Preventive Medicine<br>Canaway, Alastair; University of Warwick Warwick Medical School<br>Crepaz_Keay, David; Mental Health Foundation<br>Keown, Patrick; Newcastle University Faculty of Medical Sciences, Institute of Neuroscience; Northumberland Tyne and Wear NHS Foundation Trust,<br>Madan, Jason; University of Warwick, Warwick Medical School<br>McBride, Orla; Ulster University, Psychology<br>Moon, Graham; University of Southampton, Geography and Environment<br>Parsons, Helen; University of Warwick, Division of Health Sciences<br>Singh, Swaran; Warwick Medical School, Health Sciences Research Institute<br>Twigg, Liz; University of Portsmouth, Department of Geography
**Primary Subject Heading**:	Mental health
Secondary Subject Heading:	Health services research, Health policy
Keywords:	Adult psychiatry < PSYCHIATRY, Quality in health care < HEALTH SERVICES ADMINISTRATION & MANAGEMENT, Health policy < HEALTH SERVICES ADMINISTRATION & MANAGEMENT

SCHOLARONE™
Manuscripts

Evaluating the effects of Community Treatment Orders (CTOs) in England using the Mental Health Services Dataset (MHSDS): Protocol for a national, population-based study

Scott Weich [a], Craig Duncan [b*], Kamaldeep Bhui [c], Alastair Canaway [d], David Crepaz-Keay [e], Patrick Keown [f], Jason Madan [d], Orla McBride [g], Graham Moon [h], Helen Parsons [d], Swaran Singh [d], Liz Twigg [b]

[a] ScHARR
University of Sheffield
Sheffield S1 4DA
United Kingdom

[b] Department of Geography
University of Portsmouth
Portsmouth PO1 3HE
United Kingdom
t: +44 (0)23 92 842493
f: +44 (0)23 92 842512
e: craig.duncan@port.ac.uk
* Corresponding author

[c] Centre for Psychiatry
Barts and The London School of Medicine & Dentistry
Queen Mary University of London
London EC1M 6BQ
United Kingdom

[d] Warwick Medical School,
University of Warwick
Coventry CV4 7AL
United Kingdom

[e] Mental Health Foundation
1 London Bridge Walk
London SE1 2SX
United Kingdom

[f] Institute of Neuroscience
Newcastle University
Newcastle NE1 7RU
United Kingdom

[g] School of Psychology
Ulster University
Londonderry BT48 7JL
United Kingdom

For peer review only

For peer review only - http://bmjopen.bmj.com/site/about/guidelines.xhtml

[h] Geography and Environment
University of Southampton
Southampton SO17 1BJ
United Kingdom

Keywords: Community treatment orders; Supervised community treatment; Community mental health

Word count: 2,773 words (excluding title page, references, figures and tables)

For peer review only

For peer review only - http://bmjopen.bmj.com/site/about/guidelines.xhtml

Abstract (300 words)

Introduction

Supervised Community Treatment (SCT) for people with serious mental disorders has become accepted practice in many countries around the world. In England, SCT was adopted in 2008 in the form of Community Treatment Orders (CTOs). CTOs have been used more than expected, with significant variations between people and places. There is conflicting evidence about the effectiveness of SCT; studies based on randomised controlled trials (RCTs) have suggested few positive impacts, while those employing observational designs have been more favourable. Robust population-based studies are needed, because of the ethical challenges of undertaking further RCTs and because variation across previous studies may reflect the effects of socio-spatial context on SCT outcomes. We aim to examine spatial and temporal variation in the use, effectiveness and cost of CTOs in England through the analysis of routine administrative data.

Methods and analysis

Four years of data from the Mental Health Services Dataset (MHSDS) will be analysed using multilevel models (MMs). Models based on all patients eligible for CTOs will be used to explore variation in their use. A subset of CTO-eligible patients comprising a treatment group (CTO-patients) and a matched control group (non-CTO patients) will be used to examine variation in the association between CTO use and study outcomes. Primary outcome will be total time in hospital. Secondary outcomes will include time to first re-admission and mortality. Outputs from these models will be used to populate predictive models of health care resource use.

For peer review only - http://bmjopen.bmj.com/site/about/guidelines.xhtml

Ethics and dissemination

Ethical approval has been granted by the NHS Data Access and Advisory Group and Warwick Medical School. To ensure patient confidentiality and to meet data governance requirements, analyses will be carried out in a secure micro-data laboratory using de-identified data. Study findings will be disseminated through academic channels and shared with mental health policy makers and other stakeholders.

For peer review only

For peer review only - http://bmjopen.bmj.com/site/about/guidelines.xhtml

Strengths and limitations of this study

- This will be the largest and most complete study of its kind and the national representativeness of the study sample, deriving from routine clinical activity, is a major strength.
- The study design reflects the range of real world settings in which mental health services are delivered and patients live in England.
- The use of multilevel models will allow us to estimate variation in CTO use and outcome at individual-, area- and service-level and to establish who CTOs work for and in what circumstances.
- Study limitations include confounding by indication and bias arising from missing data within the routine administrative data source.

For peer review only

For peer review only - http://bmjopen.bmj.com/site/about/guidelines.xhtml

Introduction

Community Treatment Orders (CTOs), a form of Supervised Community Treatment (SCT), were introduced to the Mental Health Act (2007) (MHA) in England and Wales in 2008 and allow certain patients detained in hospital to continue treatment in the community following discharge under specific conditions. To be eligible for a CTO, a patient must be detained in hospital under the MHA on a Section that allows for compulsory treatment. The most common inpatient treatment orders are Sections 3 and 37, the former being a civil order and the latter a forensic, court-imposed equivalent for mentally ill offenders. A CTO provides the clinical team the power to recall the patient to hospital after discharge.

At CTO initiation (the point of discharge from inpatient care) there are two mandatory conditions, namely that the person subject to a CTO makes themselves available for assessment for its renewal and (where necessary) to be seen by an approved second opinion doctor (SOAD) for the purposes of assessing proposed treatment plan should the patient lack capacity in respect of their treatment. Discretionary conditions can also be specified which most often include treatment (i.e. medication) adherence and engagement with services (e.g. attending for appointments with professionals). CTOs do not permit compulsory (i.e. forcible) treatment in the community,[1] but those who default from these conditions may be recalled to hospital for assessment if there are grounds to suspect deterioration in their mental health and/or risk to the patient or others. Where this assessment indicates the need for further treatment, the CTO is revoked and the original treatment order applies.

There were two motivations for introducing CTOs in England and Wales[2]: to reduce 'revolving door' admissions associated with non-adherence with care by a small group of patients, and

For peer review only - http://bmjopen.bmj.com/site/about/guidelines.xhtml

to allow treatment in the least restrictive setting, in accordance with the Human Rights Act (1988). While it was originally envisaged that around 10% of eligible patients would be placed on CTOs, the figure is currently closer to 30%.[3] Since more CTOs are issued than are ended each year, the number of patients subject to CTOs has increased over time. Almost five thousand CTOs were issued in 2016-17, with use varying between demographic groups: rates of CTO use for males (11.4 per 100,000 population) were almost twice the rate for females (6.6 per 100,000 population), and the rate for Black and Black British people (60.1 per 100,000 population) was almost nine times the rate for those of White ethnicity (6.8 per 100,000 population).[4] Use also varies between National Health Service (provider) Trusts.[5,6]

SCT has been adopted in over 75 other jurisdictions around the world,[7] although it is used differently in different places. This is highlighted by its different names, including 'involuntary outpatient commitment', 'assisted outpatient treatment', 'supervised discharge', 'compulsory community treatment' and 'compulsory ambulatory treatment'.[8] In some places, SCT is initiated only by courts, while elsewhere, as in England, it remains a matter exclusively for health and social care providers, albeit within a framework prescribed by Statute and subject to legal review. There are also differences between countries in the organisation and funding of mental health services, including separation of in-patient from community mental health teams and variations in the nature and availability of care for people with severe mental illness. Generalising findings between countries is therefore problematic.

Despite widespread use, SCT remains controversial. In part, this is because patients and carers see it as coercive, raising fears of negative effects on therapeutic relationships.[9] This disquiet has been heightened by conflicting research evidence about effectiveness.[10] There

For peer review only - http://bmjopen.bmj.com/site/about/guidelines.xhtml

have been only three RCTs of CTOs, two in the US and one in the UK. None demonstrated significant benefit of CTOs in terms of hospital readmission, social functioning, quality of life, mental state or offending.[11-13] Randomised trials of CTOs face particular ethical and practical challenges (including relatively brief duration of follow-up), and these limit their utility.[14] In critically appraising the two US trials, Kisely and colleagues highlighted small sample sizes, the court-imposed nature of compulsory community treatment and the exclusion of patients with a history of violence. The OCTET trial in England experienced several methodological limitations, including the involuntary nature of the control condition, high rates of non-participation and patients who moved between trial arms after randomisation.[15-17]

Apart from the inevitable unblinding of participants and clinicians, it is impossible to disentangle the effects of legal compulsion from the vigilance and intensive care associated with CTO implementation.[8] Further trials of CTOs are unlikely, given ethical challenges inherent in randomising those who may lack insight, and who may be dangerous to themselves or others, to legal restriction versus truly voluntary care.[18]

The results of observational studies are less consistent, with some showing decreased admission rates and reduced bed use among those subject to SCT, while others do not.[2,8,10,14,19] After excluding uncontrolled studies, observational research is characterised by matched cohort designs and before and after studies, with outcomes generally (but not invariably) assessed over two years or less. More recently, findings have emerged which suggest that patients on CTOs may experience better physical health, and reduced mortality.[20,21]

For peer review only - http://bmjopen.bmj.com/site/about/guidelines.xhtml

Population-based observational studies using routine clinical and administrative data allows analysis of variation in SCT use and outcome to be studied.[10,22] We will use four years of data from the Mental Health Services Dataset (MHSDS) to address the following aims: (1) to examine spatial and temporal variation in the use of CTOs; (2) to estimate associations between CTO use and patient outcomes; (3) to explore variation in these associations between patients, places and providers, establishing when, where and for whom CTOs may be effective; and (4) to estimate the financial costs and benefits associated with CTOs.

Methods and analysis

Data sources and study outcomes

The use of CTOs in England is recorded as part of a mandatory administrative dataset, the Mental Health Services Dataset (MHSDS), formerly known as the Mental Health Minimum Dataset (MHMDS), and then the Mental Health and Learning Disabilities Dataset (MHLDDS).[23] MHSDS collates monthly returns from health service providers on all patients in contact with secondary mental health services provided and/or funded by NHS England. This includes voluntary and involuntary in-patient treatment, out-patient attendance, day treatment and other episodes of secondary mental health care.

MHSDS data are based on spells of care for individual patients. Since 2011, the dates on which specific care spells start and end have been included. Care spell dates can therefore be used to determine duration of CTO use, and hospital (re-)admissions. Four years of MHSDS data will be used covering the period 2011/12 to 2014/15, hereafter referred to as the study period. MHSDS records contain a unique patient identifier code which will be used to link patient care spells across the study period. This identifier will also be used to link patient

For peer review only - http://bmjopen.bmj.com/site/about/guidelines.xhtml

records to the Office for National Statistics' (ONS) Mortality Database, to ascertain patient deaths. Access to, and linkage of, these different administrative data sets will be facilitated by the ESRC Administrative Data Research Network.

Covariates

MHSDS provides data on a range of patient characteristics, including age, sex and ethnicity. Data on clinical characteristics are also available such as diagnosis, care clusters (groupings of patients with similar needs and problem severities)[24] and Health of the Nation Outcome Scales (outcome ratings for a range of health and social domains).[25] Additional patient characteristics such as marital, employment and accommodation status may be present in the dataset, although previous research noted high levels of missing data.[26]

MHSDS patient records also include several spatial/service setting identifiers (Table 1). These will be used to link patient records to data on factors shown by previous studies to influence mental health outcomes at local area and service provider level. Specifically, English Indices of Deprivation 2015 will provide information about local area socio-economic characteristics while ONS 2011 Census data will be used to characterise local populations, including through the use of population density and ethnic composition data.[27,28]

Data analysis

As the overarching aim of the study is to examine spatial and temporal variation in CTO use and outcome, multilevel models (MMs) will be used.[29] MMs allow variation in outcomes to be apportioned to the appropriate spatial level (e.g. between patients, local areas and service settings) as well as estimating associations with individual-, area- and service-level

For peer review only - http://bmjopen.bmj.com/site/about/guidelines.xhtml

characteristics.[30] Since the local areas that patients live in do not nest hierarchically within the health service settings where they receive care (and vice versa), cross-classified multilevel models (CCMMs) will be used (Figure 1).[31]

Analysis will be conducted in three stages. First, CCMMs based on all patients eligible for CTOs will be used to explore spatial and temporal variation in the use of CTOs. CTO use will be considered as a binary outcome (patients subject to a CTO during the study period versus those who were not) and analyses will be undertaken using logistic multilevel regression. Models will estimate the variation at each level in the risk of being on a CTO and assess the extent to which any variation in this outcome is associated with patient, local area and health service characteristics. We will also investigate the duration of time spent subject to CTO (in days). Poisson/negative binomial link function CCMMs will be used and will include offset variables to take account of different observation times for patients.[32]

Next, to examine associations between CTO use and a range of outcomes, a cohort of patients will be constructed consisting of a treatment group (those who have been on a CTO) and a matched control group (those who are eligible but who have not been on a CTO). To prevent bias arising from secular changes in clinical practice, we will frequency-match these groups according to the start date of the CTO (CTO group) and the end date of the CTO-eligible section of the Mental Health Act (non-CTO group), with tolerance limits determined after inspection of the data. As these dates are the point within the study period at which patients in both treatment groups are discharged from hospital, they represent the index date for each arm of the study. Models based on the cohort of matched patients will be used to test associations between SCT use and patient outcomes: total time spent in hospital, time to first

For peer review only - http://bmjopen.bmj.com/site/about/guidelines.xhtml

re-admission and mortality. For total time spent in hospital, count models based on Poisson or negative binomial distributions will be estimated. For time to first re-admission, discrete-time survival analysis models will be estimated. For mortality, binary response models based on the logistic distribution will be estimated. To control for confounding by indication, we will adjust analyses for the propensity of being placed on SCT,[33] with propensity scores being modelled using the pool of available variables which will include age, sex, ethnicity, diagnosis and previous admissions (where known). CCMMs based on this cohort will be used to test for the overall 'average' effect of CTO on each of the study outcomes. The models will also assess whether there is significant spatial and temporal variation around these average effects and estimate the extent to which this is associated with patient, local area and health service characteristics, including the length of time that patients are on CTOs.

Finally, we will use outputs from our CCMMs to populate predictive models of health care resource use incorporating the predicted impact of CTOs. We will model differences in treatments costs (e.g. bed days and number of contacts with mental health staff) between patients who are and are not on CTOs. We will also quantify the administrative burden of CTOs, using protocols for mandated governance processes (e.g. Mental Health Act Review Tribunals (MHRTs) and Second Opinion Approved Doctors (SOADs)) that apply to the use of CTOs. Projected costs associated with different plausible future trajectories of CTO use will be calculated and a probabilistic sensitivity analysis will be presented for each projection based on estimates of parameter uncertainty derived from the CCMMs.

CCMMs will be estimated in MLwIN using Markov Chain Monte Carlo (MCMC) Bayesian methods.[34] This approach is suited to the complexity of the MHSDS and will provide unbiased

For peer review only - http://bmjopen.bmj.com/site/about/guidelines.xhtml

measures of variation in CTO use and outcome, and associations with patient, local area and health setting characteristics. MCMC modelling diagnostics, together with measures of multilevel variation, will be used to evaluate different models to quantify where, and for whom, associations and variation occurs.[35-37]

Patient and Public Involvement

Two reference groups will provide guidance to the project to maximise its relevance and impact. The first group will consist of mental health service users and carers and will include people with lived experience of being subject to CTOs or of caring for someone who has. Participants will be recruited by the Mental Health Foundation (MHF), the largest mental health policy, research and service improvement charity in England. Recruitment will ensure diversity of age, gender and ethnicity. The second group will consist of mental health professionals and will include managers and clinicians. The reference groups will help us ensure that analyses and dissemination are relevant to the needs of stakeholders.

Dissemination

CTOs are used more often than originally anticipated in England. Although many clinicians continue to see their value[38], uncertainty and concern about their use has been expressed by regulators and legislators.[3,39] Both have called for the sort of nuanced evidence about the benefits and hazards of CTO use in real world settings which can only come from large-scale, population-based, observational research. The purposes of this study are to describe practice in respect of CTO use and duration, to estimate associations with, and spatial variation in, outcomes, and to understand where, and for whom, CTOs might be of benefit. We will also model the economic costs and benefits of current patterns of CTO use.

For peer review only - http://bmjopen.bmj.com/site/about/guidelines.xhtml

This will be the largest and most complete study of its kind in England. The national scope and representativeness of the study sample, deriving from routine clinical activity, is a major strength, as is duration of follow-up. Study limitations are those that affect observational and secondary research, particularly confounding by indication, residual confounding and bias. Issues of data quality (including missing data) are important sources of the latter. [40] The study findings will be of direct policy relevance, and we plan to share them directly with those bodies working in this area at the earliest opportunity.

For peer review only

For peer review only - http://bmjopen.bmj.com/site/about/guidelines.xhtml

Table 1: *Spatial/service setting identifiers present in the Mental Health Services Dataset (MHSDS)*

**Spatial/Service Setting Identifier**	**Description**
Lower Layer Super Output Area (LSOA)	Local Area of Residence, based on 2001 Census boundaries, typically containing 672 households and 1,614 residents
GP Practice	Primary Care Provider
NHS and Independent Sector Provider Trust	Provider of Secondary Mental Health Care
Primary Care Trust (PCT)	Commissioner of Secondary Mental Health Care

For peer review only

For peer review only - http://bmjopen.bmj.com/site/about/guidelines.xhtml

Figure 1: *An illustration of cross-classified multilevel data*

For peer review only

For peer review only - http://bmjopen.bmj.com/site/about/guidelines.xhtml

Authors' contributions

SW, KB, DC-K, PK, JM, OM, GM, HP, SS, LT had the original idea for the study and were responsible for study hypotheses, design and data specification. AC contributed to the design of the health economics work. CD was responsible for drafting the manuscript. SW has overall responsibility for the conduct of the study. All authors contributed, read and approved the final manuscript.

Funding

The work was supported by the National Institute for Health Research Health Services and Delivery Research Programme (project number 14/52/40).

Competing interests

None.

Ethics approval

The study is approved by the NHS Data Access and Advisory Group and Warwick Medical School Biomedical Research Ethics Committee.

Data sharing

Due to the data governance regulations relating to the data used in this study, no additional unpublished data is available.

For peer review only

For peer review only - http://bmjopen.bmj.com/site/about/guidelines.xhtml

References

1 Care Quality Commission. *Monitoring the Mental Health Act in 2011/12*. 2013.

2 Churchill R, Owen G, Singh S et al. *International experiences of using community treatment orders*. London: Department of Health. 2007.

3 House of Commons Health Committee. *Post-Legislative Scrutiny of the Mental Health Act 2007: First Report of Session 2013–14*. London: House of Commons. 2013.

4 NHS Digital. *Mental Health Act Statistics, Annual Figures 2016/17 Experimental Statistics*. 2017

5 Trevithick L, Carlile J, Nodiyal S, Keown P. Community treatment orders: an analysis of the first five years of use in England. *The British Journal of Psychiatry*. 2018; 212(3):175-9.

6 NHS Digital. *Inpatients formally detained in hospitals under the Mental Health Act 1983, and patients subject to supervised community treatment Uses of the Mental Health Act: Annual Statistics, 2015/16*. 2016.

7 Rugkåsa J. Effectiveness of community treatment orders: the international evidence. *The Canadian Journal of Psychiatry* 2016; 61: 15-24.

8 O'Brien AJ, McKenna BG, Kydd RR. Compulsory community mental health treatment: Literature review. *International J Nurs Stud*. 2009; 46:1245-55.

9 Light E. The epistemic challenges of CTOs. *Psychiatric Bulletin* 2014; 38: 6-8.

10 Kisely S. Assessing the effectiveness of compulsory community treatment. In Molodynski A, Rugkåsa J, Burns T eds. *Coercion in Community Mental Health Care: International Perspectives.* Oxford 2016: 75-94.

11 Swartz MS, Swanson JW, Wagner HR et al. Can involuntary outpatient commitment reduce hospital recidivism?: findings from a randomized trial with severely mentally ill individuals. *American Journal of Psychiatry* 1999; 156: 1968-1975.

12 Steadman, HJ, Gounis K, Dennis D et al. Assessing the New York City involuntary outpatient commitment pilot program. *Psychiatric Services* 2001; 52: 330-336.

13 Burns T, Rugkåsa J, Molodynski A, et al. Community treatment orders for patients with psychosis (OCTET): a randomised controlled trial. *The Lancet* 2013; 381: 1627-1633.

14 Kisely S, Campbell LA, Scott A et al. Randomized and non-randomized evidence for the effect of compulsory community and involuntary out-patient treatment on health service use: systematic review and meta-analysis. *Psychological Medicine* 2007; 37: 3-14.

15 Curtis D. OCTET does not demonstrate a lack of effectiveness for community treatment orders. *Psychiatric Bulletin* 2014; 38: 36-39.

16 Mustafa FA. On the OCTET and supervised community treatment orders. *Med Sci Law* 2014; 54: 116–117.

17 Owen A, Sood L. OCTET does not prove community treatment orders are ineffective. *The Lancet Psychiatry* 2015; 2: 373-375.

18 Treatment Advocacy Center. *No Relevance to Assisted Outpatient Treatment in the OCTET Study of English Compulsory Treatment*. 2014.

19 Maughan D, Molodynski A, Rugkåsa J et al. A systematic review of the effect of community treatment orders on service use. *Social psychiatry and psychiatric epidemiology* 2014; 49: 651-663.

For peer review only - http://bmjopen.bmj.com/site/about/guidelines.xhtml

20 Nakhost A, Perry JC, Frank D. Assessing the outcome of CTOs on management of psychiatric patients. *Can J Psychiatry*. 2012; 57:359-65.

21 Kisely S, Xiao J, Lawrence D, Jian L. Is the effect of compulsory community treatment on preventable deaths from physical disorders mediated by better access to specialized medical procedures? *Can J Psychiatry*. 2014; 59:54-8.

22 Johnson S. Can we reverse the rising tide of compulsory admissions? *Lancet* 2013; 1603-1604.

23 The NHS Information Centre. *MHMDS Statistics: Data Quality and Methodology*. 2011.

24 NHS England. Mental Health Clustering Booklet v5.0, 2016/17. https://assets.publishing.service.gov.uk/government/uploads/system/uploads/attachment_data/file/499475/Annex_B4_Mental_health_clustering_booklet.pdf

25 Royal College of Psychiatrists. Health of the Nation Outcomes Scales (HoNOS) Glossary, 2018. https://www.rcpsych.ac.uk/pdf/CALC_HoNOS2_FinalVersion_2017A.pdf

26 Weich, S., McBride, O., Twigg, L., et al. Variation in compulsory psychiatric inpatient admission in England: a cross-classified, multilevel analysis. *The Lancet Psychiatry*. 2017; 4:619-626.

27 Ministry of Housing, Communities and Local Government. *English Indices of Deprivation*. 2015.

28 Office for National Statistics. *2011 Census aggregate data*. 2016.

29 Snijders TA, Bosker R. *Multilevel Analysis: An Introduction to Basic and Advanced Multilevel Modeling (2nd Edition)*. London: Sage. 2012.

30 Merlo J. Multilevel analytical approaches in social epidemiology: measures of health variation compared with traditional measures of association. *Journal of epidemiology and community health* 2003; 57: 550-552.

31 Fielding A, Goldstein H. *Cross-Classified and Multiple Membership Structures in Models: An Introduction and Review. Research Report RR791 for DfES.* London: Department for Education and Skills; 2006.

32 Austin, PC, Stryhn H., Leckie G. and Merlo J. Measures of clustering and heterogeneity in multilevel Poisson regression analyses of rates/count data. *Statistics in Medicine 2018; 37:* 572-589.

33 Austin PC. An introduction to propensity score methods for reducing the effects of confounding in observational studies. *Multivariate behavioral research* 2011; 46: 399-424.

34 Browne WJ *MCMC Estimation in MLwiN v2.1.* Centre for Multilevel Modelling, University of Bristol. 2009.

35 Raftery AE, Lewis SM. [Practical Markov Chain Monte Carlo]: Comment: One Long Run with Diagnostics: Implementation Strategies for Markov Chain Monte Carlo. *Stat Sci* 1992; 7(4):493–7.

36 Spiegelhalter DJ, Best NG, Carlin BP, van der Linde A. Bayesian measures of model complexity and fit. *J R Stat Soc Ser B* 2002; 64: 583–639.

37 Merlo J, Ohlsson H, Lynch KF, et al. Individual and collective bodies: using measures of variance and association in contextual epidemiology. *Journal of epidemiology and community health* 2009; 63: 1043-1048.

38 DeRidder R, Manning C, Molodynski A et al. Community treatment orders in the UK 5 years on: a repeat national survey of psychiatrists. *BJPsych Bull* 2016; 40: 119-123.

39 Care Quality Commission. *Monitoring the Mental Health Act in 2012/13*. 2014.

For peer review only - http://bmjopen.bmj.com/site/about/guidelines.xhtml

40 Stuart EA, Azur M, Frangakis C et al. Multiple imputation with large data sets: a case study of the Children's Mental Health Initiative. *American journal of epidemiology* 2009; 169: 1133-9.

For peer review only

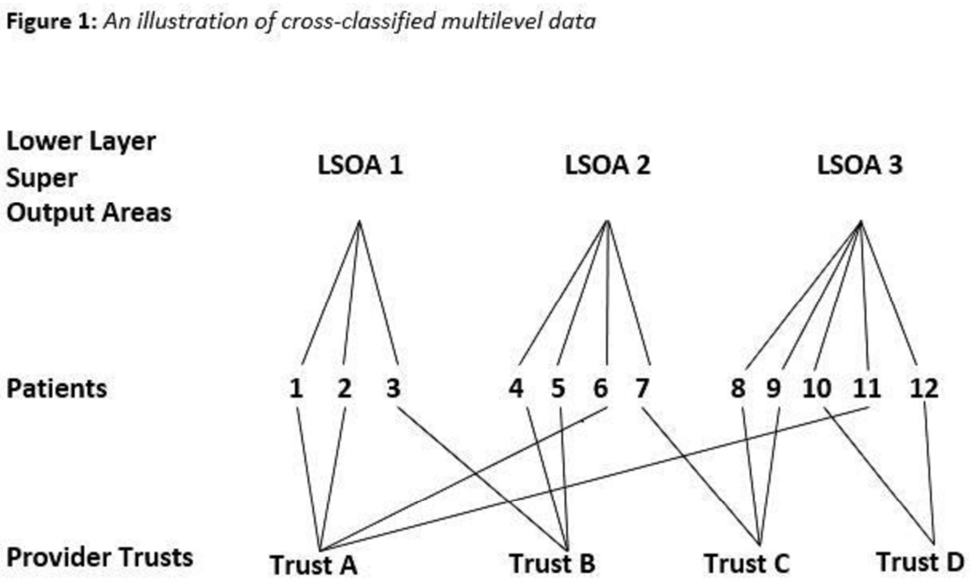

Figure 1: An illustration of cross-classified multilevel data

159x94mm (300 x 300 DPI)

BMJ Open

Evaluating the effects of Community Treatment Orders (CTOs) in England using the Mental Health Services Dataset (MHSDS): Protocol for a national, population-based study

Journal:	*BMJ Open*
Manuscript ID	bmjopen-2018-024193.R2
Article Type:	Protocol
Date Submitted by the Author:	30-Jul-2018
Complete List of Authors:	Weich, Scott; University of Sheffield, School of Health and Related Research<br>Duncan, Craig; University of Portsmouth, Geography;<br>Bhui, Kamaldeep; Barts and The London School of Medicine & Dentistry Queen Mary University of London, Centre for Psychiatry at the Wolfson Institute of Preventive Medicine<br>Canaway, Alastair; University of Warwick Warwick Medical School<br>Crepaz_Keay, David; Mental Health Foundation<br>Keown, Patrick; Newcastle University Faculty of Medical Sciences, Institute of Neuroscience; Northumberland Tyne and Wear NHS Foundation Trust,<br>Madan, Jason; University of Warwick, Warwick Medical School<br>McBride, Orla; Ulster University, Psychology<br>Moon, Graham; University of Southampton, Geography and Environment<br>Parsons, Helen; University of Warwick, Division of Health Sciences<br>Singh, Swaran; Warwick Medical School, Health Sciences Research Institute<br>Twigg, Liz; University of Portsmouth, Department of Geography
**Primary Subject Heading**:	Mental health
Secondary Subject Heading:	Health services research, Health policy
Keywords:	Adult psychiatry < PSYCHIATRY, Quality in health care < HEALTH SERVICES ADMINISTRATION & MANAGEMENT, Health policy < HEALTH SERVICES ADMINISTRATION & MANAGEMENT

SCHOLARONE™
Manuscripts

Evaluating the effects of Community Treatment Orders (CTOs) in England using the Mental Health Services Dataset (MHSDS): Protocol for a national, population-based study

Scott Weich [a], Craig Duncan [b*], Kamaldeep Bhui [c], Alastair Canaway [d], David Crepaz-Keay [e], Patrick Keown [f], Jason Madan [d], Orla McBride [g], Graham Moon [h], Helen Parsons [d], Swaran Singh [d], Liz Twigg [b]

[a] ScHARR
University of Sheffield
Sheffield S1 4DA
United Kingdom

[b] Department of Geography
University of Portsmouth
Portsmouth PO1 3HE
United Kingdom
t: +44 (0)23 92 842493
f: +44 (0)23 92 842512
e: craig.duncan@port.ac.uk
* Corresponding author

[c] Centre for Psychiatry
Barts and The London School of Medicine & Dentistry
Queen Mary University of London
London EC1M 6BQ
United Kingdom

[d] Warwick Medical School,
University of Warwick
Coventry CV4 7AL
United Kingdom

[e] Mental Health Foundation
1 London Bridge Walk
London SE1 2SX
United Kingdom

[f] Institute of Neuroscience
Newcastle University
Newcastle NE1 7RU
United Kingdom

[g] School of Psychology
Ulster University
Londonderry BT48 7JL
United Kingdom

For peer review only - http://bmjopen.bmj.com/site/about/guidelines.xhtml

[h] Geography and Environment
University of Southampton
Southampton SO17 1BJ
United Kingdom

Keywords: Community treatment orders; Supervised community treatment; Community mental health

Word count: 2,849 words (excluding title page, references, figures and tables)

For peer review only

For peer review only - http://bmjopen.bmj.com/site/about/guidelines.xhtml

Abstract (299 words)

Introduction

Supervised Community Treatment (SCT) for people with serious mental disorders has become accepted practice in many countries around the world. In England, SCT was adopted in 2008 in the form of Community Treatment Orders (CTOs). CTOs have been used more than expected, with significant variations between people and places. There is conflicting evidence about the effectiveness of SCT; studies based on randomised controlled trials (RCTs) have suggested few positive impacts, while those employing observational designs have been more favourable. Robust population-based studies are needed, because of the ethical challenges of undertaking further RCTs and because variation across previous studies may reflect the effects of socio-spatial context on SCT outcomes. We aim to examine spatial and temporal variation in the use, effectiveness and cost of CTOs in England through the analysis of routine administrative data.

Methods and analysis

Four years of data from the Mental Health Services Dataset (MHSDS) will be analysed using multilevel models (MMs). Models based on all patients eligible for CTOs will be used to explore variation in their use. A subset of CTO-eligible patients comprising a treatment group (CTO-patients) and a matched control group (non-CTO patients) will be used to examine variation in the association between CTO use and study outcomes. Primary outcome will be total time in hospital. Secondary outcomes will include time to first re-admission and mortality. Outputs from these models will be used to populate predictive models of health care resource use.

For peer review only - http://bmjopen.bmj.com/site/about/guidelines.xhtml

Ethics and dissemination

Ethical approval has been granted by the NHS Data Access and Advisory Group and Warwick University. To ensure patient confidentiality and to meet data governance requirements, analyses will be carried out in a secure micro-data laboratory using de-identified data. Study findings will be disseminated through academic channels and shared with mental health policy makers and other stakeholders.

For peer review only

For peer review only - http://bmjopen.bmj.com/site/about/guidelines.xhtml

Strengths and limitations of this study

- This will be the largest and most complete study of its kind and the national representativeness of the study sample, deriving from routine clinical activity, is a major strength.

- The study design reflects the range of real world settings in which mental health services are delivered and patients live in England.

- The use of multilevel models will allow us to estimate variation in CTO use and outcome at individual-, area- and service-level and to establish who CTOs work for and in what circumstances.

- Study limitations include confounding by indication and bias arising from missing data within the routine administrative data source.

For peer review only

For peer review only - http://bmjopen.bmj.com/site/about/guidelines.xhtml

Introduction

Community Treatment Orders (CTOs), a form of Supervised Community Treatment (SCT), were introduced to the Mental Health Act (2007) (MHA) in England and Wales in 2008 and allow certain patients detained in hospital to continue treatment in the community following discharge under specific conditions. To be eligible for a CTO, a patient must be detained in hospital under the MHA on a Section that allows for compulsory treatment. The most common inpatient treatment orders are Sections 3 and 37, the former being a civil order and the latter a forensic, court-imposed equivalent for mentally ill offenders. A CTO provides the clinical team the power to recall the patient to hospital after discharge.

At CTO initiation (the point of discharge from inpatient care) there are two mandatory conditions, namely that the person subject to a CTO makes themselves available for assessment for its renewal and (where necessary) to be seen by an approved second opinion doctor (SOAD) for the purposes of assessing proposed treatment plan should the patient lack capacity in respect of their treatment. Discretionary conditions can also be specified which most often include treatment (i.e. medication) adherence and engagement with services (e.g. attending for appointments with professionals). CTOs do not permit compulsory (i.e. forcible) treatment in the community,[1] but those who default from these conditions may be recalled to hospital for assessment if there are grounds to suspect deterioration in their mental health and/or risk to the patient or others. Where this assessment indicates the need for further treatment, the CTO is revoked and the original treatment order applies.

There were two motivations for introducing CTOs in England and Wales[2]: to reduce 'revolving door' admissions associated with non-adherence with care by a small group of patients, and

For peer review only - http://bmjopen.bmj.com/site/about/guidelines.xhtml

to allow treatment in the least restrictive setting, in accordance with the Human Rights Act (1988). While it was originally envisaged that around 10% of eligible patients would be placed on CTOs, the figure is currently closer to 30%.[3] Since more CTOs are issued than are ended each year, the number of patients subject to CTOs has increased over time. Almost five thousand CTOs were issued in 2016-17, with use varying between demographic groups: rates of CTO use for males (11.4 per 100,000 population) were almost twice the rate for females (6.6 per 100,000 population), and the rate for Black and Black British people (60.1 per 100,000 population) was almost nine times the rate for those of White ethnicity (6.8 per 100,000 population).[4] Use also varies between National Health Service (provider) Trusts.[5,6]

SCT has been adopted in over 75 other jurisdictions around the world,[7] although it is used differently in different places. This is highlighted by its different names, including 'involuntary outpatient commitment', 'assisted outpatient treatment', 'supervised discharge', 'compulsory community treatment' and 'compulsory ambulatory treatment'.[8] In some places, SCT is initiated only by courts, while elsewhere, as in England, it remains a matter exclusively for health and social care providers, albeit within a framework prescribed by Statute and subject to legal review. There are also differences between countries in the organisation and funding of mental health services, including separation of in-patient from community mental health teams and variations in the nature and availability of care for people with severe mental illness. Generalising findings between countries is therefore problematic.

Despite widespread use, SCT remains controversial. In part, this is because patients and carers see it as coercive, raising fears of negative effects on therapeutic relationships.[9] This disquiet has been heightened by conflicting research evidence about effectiveness.[10] There

For peer review only

For peer review only - http://bmjopen.bmj.com/site/about/guidelines.xhtml

have been only three RCTs of CTOs, two in the US and one in the UK. None demonstrated significant benefit of CTOs in terms of hospital readmission, social functioning, quality of life, mental state or offending.[11-13] Randomised trials of CTOs face particular ethical and practical challenges (including relatively brief duration of follow-up), and these limit their utility.[14] In critically appraising the two US trials, Kisely and colleagues highlighted small sample sizes, the court-imposed nature of compulsory community treatment and the exclusion of patients with a history of violence. The OCTET trial in England experienced several methodological limitations, including the involuntary nature of the control condition, high rates of non-participation and patients who moved between trial arms after randomisation.[15-17]

Apart from the inevitable unblinding of participants and clinicians, it is impossible to disentangle the effects of legal compulsion from the vigilance and intensive care associated with CTO implementation.[8] Further trials of CTOs are unlikely, given ethical challenges inherent in randomising those who may lack insight, and who may be dangerous to themselves or others, to legal restriction versus truly voluntary care.[18]

The results of observational studies are less consistent, with some showing decreased admission rates and reduced bed use among those subject to SCT, while others do not.[2,8,10,14,19] After excluding uncontrolled studies, observational research is characterised by matched cohort designs and before and after studies, with outcomes generally (but not invariably) assessed over two years or less. More recently, findings have emerged which suggest that patients on CTOs may experience better physical health, and reduced mortality.[20,21]

For peer review only - http://bmjopen.bmj.com/site/about/guidelines.xhtml

Population-based observational studies using routine clinical and administrative data allows analysis of variation in SCT use and outcome to be studied.[10,22] We will use four years of data from the Mental Health Services Dataset (MHSDS) to address the following aims: (1) to examine spatial and temporal variation in the use of CTOs; (2) to estimate associations between CTO use and patient outcomes; (3) to explore variation in these associations between patients, places and providers, establishing when, where and for whom CTOs may be effective; and (4) to estimate the financial costs and benefits associated with CTOs.

Methods and analysis

Data sources and study outcomes

The use of CTOs in England is recorded as part of a mandatory administrative dataset, the Mental Health Services Dataset (MHSDS), formerly known as the Mental Health Minimum Dataset (MHMDS), and then the Mental Health and Learning Disabilities Dataset (MHLDDS).[23] MHSDS collates monthly returns from health service providers on all patients in contact with secondary mental health services provided and/or funded by NHS England. This includes voluntary and involuntary in-patient treatment, out-patient attendance, day treatment and other episodes of secondary mental health care.

MHSDS data are based on spells of care for individual patients. Since 2011, the dates on which specific care spells start and end have been included. Care spell dates can therefore be used to determine duration of CTO use, and hospital (re-)admissions. Four years of MHSDS data will be used covering the period 2011/12 to 2014/15, hereafter referred to as the study period. MHSDS records contain a unique patient identifier code which will be used to link patient care spells across the study period. This identifier will also be used to link patient

For peer review only - http://bmjopen.bmj.com/site/about/guidelines.xhtml

records to the Office for National Statistics' (ONS) Mortality Database, to ascertain patient deaths. Access to, and linkage of, these different administrative data sets will be facilitated by the ESRC Administrative Data Research Network.

Covariates

MHSDS provides data on a range of patient characteristics, including age, sex and ethnicity. Data on clinical characteristics are also available such as diagnosis, care clusters (groupings of patients with similar needs and problem severities)[24] and Health of the Nation Outcome Scales (outcome ratings for a range of health and social domains).[25] Additional patient characteristics such as marital, employment and accommodation status may be present in the dataset, although previous research noted high levels of missing data.[26]

MHSDS patient records also include several spatial/service setting identifiers (Table 1). These will be used to link patient records to data on factors shown by previous studies to influence mental health outcomes at local area and service provider level. Specifically, English Indices of Deprivation 2015 will provide information about local area socio-economic characteristics while ONS 2011 Census data will be used to characterise local populations, including through the use of population density and ethnic composition data.[27,28]

Data analysis

As the overarching aim of the study is to examine spatial and temporal variation in CTO use and outcome, multilevel models (MMs) will be used.[29] MMs allow variation in outcomes to be apportioned to the appropriate spatial level (e.g. between patients, local areas and service settings) as well as estimating associations with individual-, area- and service-level

For peer review only - http://bmjopen.bmj.com/site/about/guidelines.xhtml

characteristics.[30] Since the local areas that patients live in do not nest hierarchically within the health service settings where they receive care (and vice versa), cross-classified multilevel models (CCMMs) will be used (Figure 1).[31]

Analysis will be conducted in three stages. First, CCMMs based on all patients eligible for CTOs will be used to explore spatial and temporal variation in the use of CTOs. CTO use will be considered as a binary outcome (patients subject to a CTO during the study period versus those who were not) and analyses will be undertaken using logistic multilevel regression. Models will estimate the variation at each level in the risk of being on a CTO and assess the extent to which any variation in this outcome is associated with patient, local area and health service characteristics. We will also investigate the duration of time spent subject to CTO (in days). Poisson/negative binomial link function CCMMs will be used and will include offset variables to take account of different observation times for patients.[32]

Next, to examine associations between CTO use and a range of outcomes, a cohort of patients will be constructed consisting of a treatment group (those who have been on a CTO) and a matched control group (those who are eligible but who have not been on a CTO). To prevent bias arising from secular changes in clinical practice, we will frequency-match these groups according to the start date of the CTO (CTO group) and the end date of the CTO-eligible section of the Mental Health Act (non-CTO group), with tolerance limits determined after inspection of the data. As these dates are the point within the study period at which patients in both treatment groups are discharged from hospital, they represent the index date for each arm of the study. Models based on the cohort of matched patients will be used to test associations between SCT use and patient outcomes: total time spent in hospital, time to first

For peer review only - http://bmjopen.bmj.com/site/about/guidelines.xhtml

re-admission and mortality. For total time spent in hospital, count models based on Poisson or negative binomial distributions will be estimated. For time to first re-admission, discrete-time survival analysis models will be estimated. For mortality, binary response models based on the logistic distribution will be estimated. To control for confounding by indication, we will adjust analyses for the propensity of being placed on SCT,[33] with propensity scores being modelled using the pool of available variables which will include age, sex, ethnicity, diagnosis and previous admissions (where known). CCMMs based on this cohort will be used to test for the overall 'average' effect of CTO on each of the study outcomes. The models will also assess whether there is significant spatial and temporal variation around these average effects and estimate the extent to which this is associated with patient, local area and health service characteristics, including the length of time that patients are on CTOs.

Finally, we will use outputs from our CCMMs to populate predictive models of health care resource use incorporating the predicted impact of CTOs. We will model differences in treatments costs (e.g. bed days and number of contacts with mental health staff) between patients who are and are not on CTOs. We will also quantify the administrative burden of CTOs, using protocols for mandated governance processes (e.g. Mental Health Act Review Tribunals (MHRTs) and Second Opinion Approved Doctors (SOADs)) that apply to the use of CTOs. Projected costs associated with different plausible future trajectories of CTO use will be calculated and a probabilistic sensitivity analysis will be presented for each projection based on estimates of parameter uncertainty derived from the CCMMs.

CCMMs will be estimated in MLwIN using Markov Chain Monte Carlo (MCMC) Bayesian methods.[34] This approach is suited to the complexity of the MHSDS and will provide unbiased

For peer review only - http://bmjopen.bmj.com/site/about/guidelines.xhtml

measures of variation in CTO use and outcome, and associations with patient, local area and health setting characteristics. MCMC modelling diagnostics, together with measures of multilevel variation, will be used to evaluate different models to quantify where, and for whom, associations and variation occurs.[35-37]

Patient and Public Involvement

Two reference groups will provide guidance to the project to maximise its relevance and impact. The first group will consist of mental health service users and carers and will include people with lived experience of being subject to CTOs or of caring for someone who has. Participants will be recruited by the Mental Health Foundation (MHF), the largest mental health policy, research and service improvement charity in England. Recruitment will ensure diversity of age, gender and ethnicity. The second group will consist of mental health professionals and will include managers and clinicians. The reference groups will help us ensure that analyses and dissemination are relevant to the needs of stakeholders.

Ethics and Dissemination

Ethical approval has been granted by the NHS Data Access and Advisory Group and the University of Warwick's Biomedical and Scientific Research Ethics Committee (BSREC Ref: REGO-2015-1623). To ensure patient confidentiality and to meet data governance requirements, analyses will be carried out in a secure micro-data laboratory at the Office for National Statistics using de-identified data. Reporting of the study will be consistent with the STROBE guidelines.[38]

CTOs are used more often than originally anticipated in England. Although many clinicians

For peer review only - http://bmjopen.bmj.com/site/about/guidelines.xhtml

continue to see their value[39], uncertainty and concern about their use has been expressed by regulators and legislators.[3,40] Both have called for the sort of nuanced evidence about the benefits and hazards of CTO use in real world settings which can only come from large-scale, population-based, observational research. The purposes of this study are to describe practice in respect of CTO use and duration, to estimate associations with, and spatial variation in, outcomes, and to understand where, and for whom, CTOs might be of benefit. We will also model the economic costs and benefits of current patterns of CTO use.

This will be the largest and most complete study of its kind in England. The national scope and representativeness of the study sample, deriving from routine clinical activity, is a major strength, as is duration of follow-up. Study limitations are those that affect observational and secondary research, particularly confounding by indication, residual confounding and bias. Issues of data quality (including missing data) are important sources of the latter. [41] The study findings will be of direct policy relevance, and we plan to share them directly with those bodies working in this area at the earliest opportunity.

For peer review only

Table 1: *Spatial/service setting identifiers present in the Mental Health Services Dataset (MHSDS)*

**Spatial/Service Setting Identifier**	**Description**
Lower Layer Super Output Area (LSOA)	Local Area of Residence, based on 2001 Census boundaries, typically containing 672 households and 1,614 residents
GP Practice	Primary Care Provider
NHS and Independent Sector Provider Trust	Provider of Secondary Mental Health Care
Primary Care Trust (PCT)	Commissioner of Secondary Mental Health Care

For peer review only

For peer review only - http://bmjopen.bmj.com/site/about/guidelines.xhtml

Figure 1: *An illustration of cross-classified multilevel data*

For peer review only

For peer review only - http://bmjopen.bmj.com/site/about/guidelines.xhtml

Authors' contributions

SW, KB, DC-K, PK, JM, OM, GM, HP, SS, LT had the original idea for the study and were responsible for study hypotheses, design and data specification. AC contributed to the design of the health economics work. CD was responsible for drafting the manuscript. SW has overall responsibility for the conduct of the study. All authors contributed, read and approved the final manuscript.

Funding

The work was supported by the National Institute for Health Research Health Services and Delivery Research Programme (project number 14/52/40).

Competing interests

None.

Ethics approval

The study is approved by the NHS Data Access and Advisory Group and the University of Warwick's Biomedical and Scientific Research Ethics Committee (BSREC Ref: REGO-2015-1623).

Data sharing

Due to the data governance regulations relating to the data used in this study, no additional unpublished data is available.

For peer review only

References

1 Care Quality Commission. *Monitoring the Mental Health Act in 2011/12*. 2013.

2 Churchill R, Owen G, Singh S et al. *International experiences of using community treatment orders*. London: Department of Health. 2007.

3 House of Commons Health Committee. *Post-Legislative Scrutiny of the Mental Health Act 2007: First Report of Session 2013–14*. London: House of Commons. 2013.

4 NHS Digital. *Mental Health Act Statistics, Annual Figures 2016/17 Experimental Statistics*. 2017

5 Trevithick L, Carlile J, Nodiyal S, Keown P. Community treatment orders: an analysis of the first five years of use in England. *The British Journal of Psychiatry*. 2018; 212(3):175-9.

6 NHS Digital. *Inpatients formally detained in hospitals under the Mental Health Act 1983, and patients subject to supervised community treatment Uses of the Mental Health Act: Annual Statistics, 2015/16*. 2016.

7 Rugkåsa J. Effectiveness of community treatment orders: the international evidence. *The Canadian Journal of Psychiatry* 2016; 61: 15-24.

8 O'Brien AJ, McKenna BG, Kydd RR. Compulsory community mental health treatment: Literature review. *International J Nurs Stud*. 2009; 46:1245-55.

9 Light E. The epistemic challenges of CTOs. *Psychiatric Bulletin* 2014; 38: 6-8.

10 Kisely S. Assessing the effectiveness of compulsory community treatment. In Molodynski A, Rugkåsa J, Burns T eds. *Coercion in Community Mental Health Care: International Perspectives.* Oxford 2016: 75-94.

11 Swartz MS, Swanson JW, Wagner HR et al. Can involuntary outpatient commitment reduce hospital recidivism?: findings from a randomized trial with severely mentally ill individuals. *American Journal of Psychiatry* 1999; 156: 1968-1975.

12 Steadman, HJ, Gounis K, Dennis D et al. Assessing the New York City involuntary outpatient commitment pilot program. *Psychiatric Services* 2001; 52: 330-336.

13 Burns T, Rugkåsa J, Molodynski A, et al. Community treatment orders for patients with psychosis (OCTET): a randomised controlled trial. *The Lancet* 2013; 381: 1627-1633.

14 Kisely S, Campbell LA, Scott A et al. Randomized and non-randomized evidence for the effect of compulsory community and involuntary out-patient treatment on health service use: systematic review and meta-analysis. *Psychological Medicine* 2007; 37: 3-14.

15 Curtis D. OCTET does not demonstrate a lack of effectiveness for community treatment orders. *Psychiatric Bulletin* 2014; 38: 36-39.

16 Mustafa FA. On the OCTET and supervised community treatment orders. *Med Sci Law* 2014; 54: 116–117.

17 Owen A, Sood L. OCTET does not prove community treatment orders are ineffective. *The Lancet Psychiatry* 2015; 2: 373-375.

18 Treatment Advocacy Center. *No Relevance to Assisted Outpatient Treatment in the OCTET Study of English Compulsory Treatment*. 2014.

19 Maughan D, Molodynski A, Rugkåsa J et al. A systematic review of the effect of community treatment orders on service use. *Social psychiatry and psychiatric epidemiology* 2014; 49: 651-663.

For peer review only - http://bmjopen.bmj.com/site/about/guidelines.xhtml

20 Nakhost A, Perry JC, Frank D. Assessing the outcome of CTOs on management of psychiatric patients. *Can J Psychiatry*. 2012; 57:359-65.

21 Kisely S, Xiao J, Lawrence D, Jian L. Is the effect of compulsory community treatment on preventable deaths from physical disorders mediated by better access to specialized medical procedures? *Can J Psychiatry*. 2014; 59:54-8.

22 Johnson S. Can we reverse the rising tide of compulsory admissions? *Lancet* 2013; 1603-1604.

23 The NHS Information Centre. *MHMDS Statistics: Data Quality and Methodology*. 2011.

24 NHS England. Mental Health Clustering Booklet v5.0, 2016/17. https://assets.publishing.service.gov.uk/government/uploads/system/uploads/attachment_data/file/499475/Annex_B4_Mental_health_clustering_booklet.pdf

25 Royal College of Psychiatrists. Health of the Nation Outcomes Scales (HoNOS) Glossary, 2018. https://www.rcpsych.ac.uk/pdf/CALC_HoNOS2_FinalVersion_2017A.pdf

26 Weich, S., McBride, O., Twigg, L., et al. Variation in compulsory psychiatric inpatient admission in England: a cross-classified, multilevel analysis. *The Lancet Psychiatry*. 2017; 4:619-626.

27 Ministry of Housing, Communities and Local Government. *English Indices of Deprivation*. 2015.

28 Office for National Statistics. *2011 Census aggregate data*. 2016.

29 Snijders TA, Bosker R. *Multilevel Analysis: An Introduction to Basic and Advanced Multilevel Modeling (2nd Edition)*. London: Sage. 2012.

30 Merlo J. Multilevel analytical approaches in social epidemiology: measures of health variation compared with traditional measures of association. *Journal of epidemiology and community health* 2003; 57: 550-552.

31 Fielding A, Goldstein H. *Cross-Classified and Multiple Membership Structures in Models: An Introduction and Review. Research Report RR791 for DfES.* London: Department for Education and Skills; 2006.

32 Austin, PC, Stryhn H., Leckie G. and Merlo J. Measures of clustering and heterogeneity in multilevel Poisson regression analyses of rates/count data. *Statistics in Medicine 2018; 37:* 572-589.

33 Austin PC. An introduction to propensity score methods for reducing the effects of confounding in observational studies. *Multivariate behavioral research* 2011; 46: 399-424.

34 Browne WJ *MCMC Estimation in MLwiN v2.1.* Centre for Multilevel Modelling, University of Bristol. 2009.

35 Raftery AE, Lewis SM. [Practical Markov Chain Monte Carlo]: Comment: One Long Run with Diagnostics: Implementation Strategies for Markov Chain Monte Carlo. *Stat Sci* 1992; 7(4):493–7.

36 Spiegelhalter DJ, Best NG, Carlin BP, van der Linde A. Bayesian measures of model complexity and fit. *J R Stat Soc Ser B* 2002; 64: 583–639.

37 Merlo J, Ohlsson H, Lynch KF, et al. Individual and collective bodies: using measures of variance and association in contextual epidemiology. *Journal of epidemiology and community health* 2009; 63: 1043-1048.

38 von Elm E, Altman DG, Egger M, Pocock SJ, Gøtzsche PC, Vandenbroucke JP, STROBE Initiative. The Strengthening the Reporting of Observational Studies in Epidemiology (STROBE)

For peer review only - http://bmjopen.bmj.com/site/about/guidelines.xhtml

statement: guidelines for reporting observational studies. Ann Intern Med 2007 Oct 16;147(8):573-577.

39 DeRidder R, Manning C, Molodynski A et al. Community treatment orders in the UK 5 years on: a repeat national survey of psychiatrists. *BJPsych Bull* 2016; 40: 119-123.

40 Care Quality Commission. *Monitoring the Mental Health Act in 2012/13*. 2014.

41 Stuart EA, Azur M, Frangakis C et al. Multiple imputation with large data sets: a case study of the Children's Mental Health Initiative. *American journal of epidemiology* 2009; 169: 1133-9.

For peer review only

For peer review only - http://bmjopen.bmj.com/site/about/guidelines.xhtml

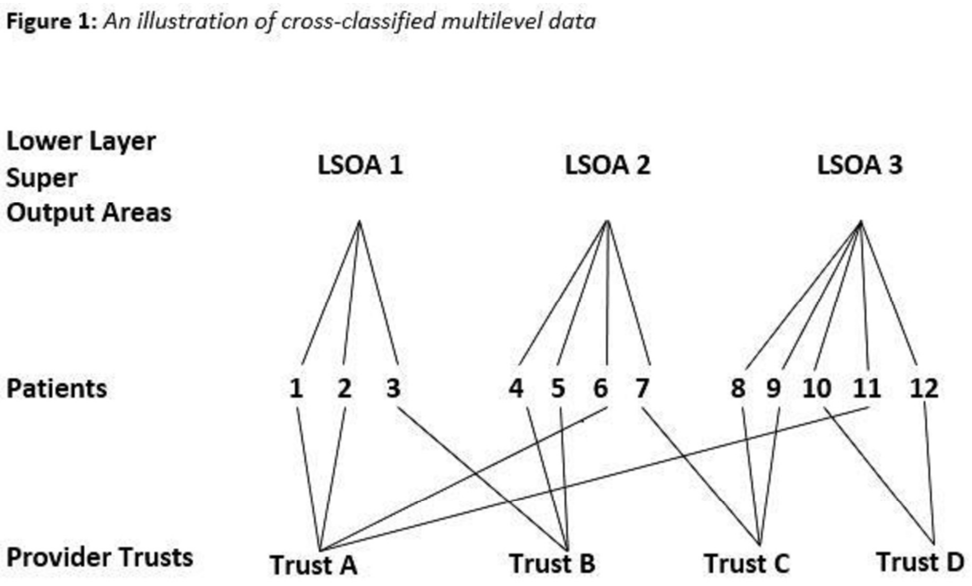

Figure 1: An illustration of cross-classified multilevel data

159x94mm (300 x 300 DPI)
